# Treatment of Cachexia in Oncology

**DOI:** 10.4103/0973-1075.73644

**Published:** 2010

**Authors:** EM Tazi, H Errihani

**Affiliations:** Department of Medical Oncology, National Institute of Oncology, Rabat, Morocco

**Keywords:** Cachexia, Oncology, Treatment

## Abstract

Cachexia is a complex metabolic syndrome associated with many chronic or end-stage diseases, especially cancer, and is characterized by loss of muscle with or without loss of fat mass. The management of cachexia is a complex challenge that should address the different causes underlying this clinical event with an integrated or multimodal treatment approach targeting the different factors involved in its pathophysiology. The purpose of this article was to review the current medical treatment of cancer-related cachexia, in particular focusing on combination therapy and ongoing research. Among the treatments proposed in the literature for cancer-related cachexia, some proved to be ineffective, namely, cyproheptadine, hydrazine, metoclopramide, and pentoxifylline. Among effective treatments, progestagens are currently considered the best available treatment option for cancer-related cachexia, and they are the only drugs approved in Europe. Drugs with a strong rationale that have failed or have not shown univocal results in clinical trials so far include eicosapentaenoic acid, cannabinoids, bortezomib, and anti-TNF-alpha MoAb. Several emerging drugs have shown promising results but are still under clinical investigation (thalidomide, selective cox-2 inhibitors, ghrelin mimetics, insulin, oxandrolone, and olanzapine). To date, despite several years of coordinated efforts in basic and clinical research, practice guidelines for the prevention and treatment of cancer-related muscle wasting are lacking, mainly because of the multifactorial pathogenesis of the syndrome. From all the data presented, one can speculate that one single therapy may not be completely successful in the treatment of cachexia. From this point of view, treatments involving different combinations are more likely to be successful.

## INTRODUCTION

Cachexia is a complex metabolic syndrome associated with underlying illness and characterized by loss of muscle with or without loss of fat mass. Cachexia can occur as part of many chronic or end-stage diseases such as infections, cancer, AIDS, congestive heart failure, chronic renal failure, rheumatoid arthritis, tuberculosis, and chronic obstructive pulmonary disease. The prominent clinical feature of cachexia is weight loss in adults (corrected for fluid retention). Anorexia, inflammation, insulin resistance, and increased muscle protein breakdown are frequently associated with cachexia. Cachexia is distinct from starvation, age-related loss of muscle mass, primary depression, malabsorption, and hyperthyroidism, and is associated with increased morbidity.[1] Cachexia defines a distinct clinical syndrome where the activation of proinflammatory cytokines has a direct effect on muscle metabolism and anorexia.[2] Multiple mechanisms appear to be involved in the development of cachexia, including anorexia, decreased physical activity, decreased secretion of host anabolic hormones, and altered host metabolic response with abnormalities in protein, lipid, and carbohydrate metabolism. Basically, cachexia is dependent from cytokine-driven dysregulation of the peripheral signals (mainly leptin, ghrelin, and serotonin), reaching the brain hypothalamic region, which plays a central role in balancing the orexigenic and anorexigenic signals, leading to decreased food intake and increased resting energy expenditure (REE). Indeed, an increased REE may contribute to the loss of body weight in cachectic patients and may explain the increased oxidation of fat tissue. Futile energy-consuming cycles, such as the Cori cycle, may also play a role in the increased energy demand. Unlike starvation, body weight loss in cachectic patients arises mainly from loss of muscle mass, characterized by increased catabolism of skeletal muscle and decreased protein synthesis.

### Management of cancer cachexia

The best management of cancer cachexia is to cure the cancer as this will completely reverse the cachexia syndrome. Unfortunately, this remains an infrequent achievement in adults with advanced solid tumors. A second option could be to counteract weight loss by increasing nutritional intake, but since in the majority of cachectic patients, anorexia in only a part of the problem, nutrition as a unimodal therapy was not completely able to reverse the wasting associated to cachexia. Indeed, a large number of randomized controlled trials of nutritional intervention did not show a significant benefit with regard to weight change or quality of life. These results have led to attempts to manipulate the process of cachexia with a variety of pharmacological agents, with the main purpose of providing symptomatic improvement. To date, however, despite several years of coordinated efforts in basic and clinical research, practice guidelines for the prevention and treatment of cancer-related muscle wasting are lacking, mainly because of the multifactorial pathogenesis of the syndrome.[3]

Table 1 summarizes the currently available different therapeutic approaches, emerging drugs, and future trends for the treatment of Cancer-Related Anorexia–Cachexia Syndrome (CACS).

**Table 1:** Currently available different therapeutic approaches, emerging drugs, and future trends for the treatment of cancer-related anorexia–cachexia syndrome

	Level of evidence
Drugs commonly used	
Progestagens: megestrol acetate/Medroxyprogesterone acetate	1
Corticosteroids	1
Drugs with a strong rationale that failed or did not show univocal results in clinical trials	
Omega-3 fatty acids—EPA	1
Cannabinoids (dronabinol)	1
Bortezomib	3
Emerging drugs with some effective results but still under clinical evaluation	
Thalidomide	2
Ghrelin	2
COX-2 inhibitors	2
Insulin	2
BCAA	NA
Oxandrolone	2
Future trends	
Melanocortin antagonists	NA
β2 agonists (formoterol)	NA
Anti-myostatin peptibody	NA
Anti-IL-6	NA
SARMs	NA

### Effective treatments

#### Progestagens

Progestagens, that is, Medroxyprogesterone Acetate (MPA) and Megestrol Acetate (MA) are currently considered the best available treatment option for CACS, and they are approved in Europe for treatment of cancer- and AIDS-related cachexia. However, progestational agents are nonetheless limited in their ability to treat cancer cachexia. Fewer than 30% of patients treated with MA experience short-term appetite stimulation,[4] and although weight and appetite improve, there is no demonstrated improvement in quality of life or survival.[5,6] The proposed mechanism of action of progestagens in CACS has not been completely elucidated. It may be related to glucocorticoid activity, making these drugs similar to corticosteroids. Moreover, there is evidence that progestagens may stimulate appetite by inducing the release of neuropeptide Y in the CNS (ventromedial hypothalamus). Furthermore, they act, at least in part, by downregulating the synthesis and release of proinflammatory cytokines.[7] Historically, Mantovani and al study,[8] including only a small number of patients, has been one of the first to demonstrate the clinical effectiveness of MA and correlate it to the downregulation and reduced release of proinflammatory cytokines. Eleven male patients with head and neck cancer in advanced stage were enrolled in the study and received MA at a dose of 320 mg/day for two months during the interval of chemotherapy treatment. Weight and appetite increased significantly, and the Spitzer’s quality of life improved significantly, whereas proinflammatory cytokines levels decreased significantly.

In a subsequent *in vitro* study,[9] they showed that MPA was also able to reduce the production of cytokine and serotonin by PBMC of advanced stage cancer patients. Among progestagens, megestrol has been the drug most widely studied for its effect on CAC, with eight randomized, double-blind, placebo-controlled trials compared with medroxyprogesterone (two placebo-controlled studies).[7] Simons *et al*., carried out a randomized placebo-controlled study[10] to investigate the effects of MPA on food intake, body composition, and REE on 54 patients with non-hormone-sensitive cancer. Patients received either MPA, 500 mg, or placebo twice daily for 12 weeks. Compared with placebo, 12 weeks of MPA led to an increase in energy intake that was significantly associated with an increase in fat mass. Fat-free mass was not significantly influenced and REE increased in the MPA arm. Berenstein and Ortiz[11] undertook a literature review to evaluate the efficacy, effectiveness, and safety of MA in palliating anorexia–cachexia syndrome in patients with cancer, AIDS, and other underlying pathologies. Thirty trials were included in the original review, four new trials were identified for this update, but only two met the inclusion criteria (4123 + 703 patients). Twenty-two trials compared MA at different doses with placebo, five compared different doses of MA versus other drugs, two compared MA with other drugs and placebo, and five compared different doses of MA. For all patient conditions, meta-analysis showed a benefit of MA compared with placebo, particularly with regard to appetite improvement and weight gain in cancer patients. Analyzing quality of life, clinical and statistical heterogeneity was found and discussed. There was insufficient information to define the optimal dose of MA. In summary, this review demonstrates that MA improves appetite and weight gain in patients with cancer while no overall conclusion about quality of life could be drawn due to heterogeneity. Femia *et al*.,[12] carried out a study to assess whether MA delivered by nanoscrystal oral suspension could have the potential to improve outcomes in cachexia. MA nanocrystal oral suspension was designed to optimize drug delivery and improve bioavailability, enhancing the performance of drugs with poor water solubility. It was shown that by rapidly increasing plasma MA concentrations, this formulation could have the potential to produce a more rapid clinical response. It was approved by FDA for the treatment of AIDS-related cachexia, and it is currently under evaluation for approval in cancer cachexia. Comprehensively, progestagens have shown to be effective as regards body weight increase (mainly water and fat mass) and improvement of cenestesis and quality of life, but have not proved to be effective in increasing lean body mass (LBM), which is a critical target in the treatment of cancer cachexia. Moreover, no benefit on oxidative stress (OS) has been reported.

#### Corticosteroids

Among orexigenic agents, corticosteroids are widely used. In randomized controlled studies, they have been shown to improve appetite and quality of life compared with placebo.[13,14] MA and corticosteroids seem equally effective, although for long-term use, corticosteroids have more side effects:[5] protein breakdown, insulin resistance, water retention, and adrenal suppression are the most serious adverse effects of corticosteroid long-term treatment. Therefore, corticosteroids are not suitable for long-term use and should be used during the pre-terminal phase of cachexia.

### Drugs with a strong rationale that have failed or have not shown univocal results in clinical trials so far

Drugs able to inhibit the synthesis and/or release of cytokines (i.e. eicosapentaenoic acid (EPA), melatonin, etc.), the cytokine action (i.e. anti-cytokine antibodies, anti-inflammatory cytokines interleukin (IL)-12, and IL- 15), and drugs able to inhibit the proteasome activity (i.e. bortezomib) have been tested in experimental models of cachexia, with some positive results. Unfortunately, most clinical trials in humans have provided limited or disappointing results. N-3 fatty acids, especially EPA, may have anticachectic properties. The first study by Fearon[15] suggests that if taken in sufficient quantity, only the omega-3 fatty acid enriched energy and protein dense supplement results in net gain of weight, lean tissue, and improved quality of life. Later, a study by Jatoi *et al*.,[16] comparing EPA supplement versus MA versus both in cachectic cancer patients showed that the EPA supplement either alone or in combination with MA does not improve weight or appetite more than MA alone. A third study, carried out by Fearon,[17] compared EPA diethyl ester with placebo in cachectic cancer patients. The results of the trial indicated no statistically significant benefit from single agent EPA in the treatment of cancer cachexia. The Cochrane meta-analysis published in 2007[18] concluded that there were insufficient data to establish whether oral EPA was better than placebo. Furthermore, comparisons of EPA combined with a protein energy supplementation versus a protein energy supplementation (without EPA) in the presence of an appetite stimulant (Megestrol Acetate) provided no evidence that EPA improves symptoms associated with the cachexia syndrome often seen in patients with advanced cancer. Among the potentially effective approaches against CACS, there are cannabinoids but unfortunately two different randomized studies carried out by Jatoi *et al*.,[6] and Strasser *et al*.,[19] have failed to show better results as compared to MA or placebo, respectively. Bortezomib, an NF-kB and ubiquitin-proteasome inhibitor, although potentially promising, in preliminary results published by Jatoi[20] showed negligible favorable effects on cancer-associated weight loss in patients with metastatic pancreatic cancer. The authors concluded that further study of bortezomib specifically in this setting and for this indication were not warranted. The approach with an anti-TNF-alpha MoAb (infliximab) was shown to be ineffective. Indeed, the clinical study carried out by Wiedenmann *et al*.,[21] showed that the addition of infliximab to gemcitabine to treat cachexia in advanced pancreatic cancer patients was not associated with statistically significant differences in safety or efficacy when compared to placebo.

### Emerging drugs with some effective results but still under clinical evaluation

#### Thalidomide

Thalidomide has multiple immunomodulatory and antiinflammatory properties; its inhibitory effect on TNF-α and IL-6 production may be responsible for its apparent anticachectic activity. Thus, thalidomide has been used for treatment of cachexia associated with acquired immunodeficiency syndrome, tuberculosis, and cancer. In the current literature, there are few studies that have assessed the anabolic effects of thalidomide in gastrointestinal cancer cachexia. Gordon *et al*.,[22] undertook a randomized placebo-controlled study to assess the safety and efficacy of thalidomide in attenuating weight loss in patients with cachexia secondary to advanced pancreatic cancer and concluded that thalidomide was well tolerated but it was only able to attenuate loss of weight and LBM in patients with cachexia due to advanced pancreatic cancer.

#### Selective COX-2 inhibitors

Research on experimental animal models has shown that non-steroidal anti-inflammatory drugs, including cyclooxygenase-2 (COX-2) inhibitors, may palliate cachexia through the suppression of systemic inflammation. Lai *et al*.,[23] carried out a placebo-controlled study with celecoxib on 11 cachectic patients with head and neck or gastrointestinal cancer. Patients receiving celecoxib showed a statistically significant increase in weight and body mass index compared with placebo. The results of this pilot study are consistent with prior animal experiments and should stimulate larger clinical trials investigating the role of COX-2 inhibitors in the treatment of cancer cachexia. Indeed, more studies are needed to confirm the findings of this pilot study.

#### Ghrelin mimetic with orexigenic and anabolic activity

Recently, much research interest has focused on ghrelin, a 28 amino acid peptide produced by the P/D1 cells of the stomach. Not only does ghrelin stimulate GH secretion (via the GH secretagogue-1a (GHS-1a) receptor) but it also promotes food intake (via the orexigenic NPY system) and decreases sympathetic nerve activity. Synthetic human ghrelin has been shown to improve muscle wasting and functional capacity in patients with cardiopulmonary-associated cachexia and to improve energy intake in anorexic cancer patients. Based on the animal studies and short-term human trials, there appears to be much promise for further studies to investigate the use of ghrelin and GHS-R agonists for the treatment of cachexia caused by multiple underlying conditions. Significant questions remain to be answered, however, before its widespread use, most prominently whether the gains produced by GHS R agonists maintain safety and efficacy with long-term use in human diseases. Clearly, more long-term research is needed.[24]

#### Emergence of ghrelin as a treatment for cachexia syndrome

of ghrelin to humans with cachexia has shown no univocal efficacy in increasing food intake with single dose intravenous administration.[25,26] In a study carried out by Strasser *et al*.,[25] 21 adult patients were randomized to receive ghrelin on days 1 and 8 and placebo on days 4 and 11 or vice versa, given intravenously over a 60-min period before lunch: 10 received 2 mg kg/1 (lower dose) ghrelin and 11 received 8 mg kg/1 (upper-dose) ghrelin. Nutritional intake and eating-related symptoms, measured to explore preliminary efficacy, did not differ between ghrelin and placebo. Ghrelin is well tolerated and safe in patients with advanced cancer. For safety, tolerance, and patient preference for treatment, no difference was observed between the lower and upper-dose group.

Neary *et al*.,[26] carried out an acute, randomized, placebo-controlled, cross-over clinical trial to determine whether ghrelin (5 pmol/kg/min for 180 min i.c.) stimulates appetite in cancer patients with anorexia. Seven cancer patients who reported loss of appetite were recruited from oncology clinics at Charing Cross Hospital. A marked increase in energy intake was observed with ghrelin infusion compared with saline control, and every patient reported food intake increase. The meal appreciation score was greater by 28.8% with ghrelin treatment. No side effects were observed. Garcia *et al*.,[27] carried out a study on GHS-R agonist RC-1291 (Anamorelin; Sapphire Therapeutics, Bridgewater, NJ, USA), a small-molecule orally active compound. The compound was administered in a randomized, placebo-controlled trial over a 12-week period to 81 subjects with a variety of cancers (predominantly lung cancer). Over this 12-week course, RC-1291 produced an improvement in total body mass and a trend toward increased lean mass. A measurement of quality of life – an important consideration for any late-term cancer treatment – was unchanged between the groups receiving RC-1291 and placebo. However, it has to be underlined that the above studies are small phase I and phase II trials, and therefore, their result should be treated with caution. Further controlled randomized studies are warranted before the use of ghrelin can be translated into clinical practice.

#### Insulin

Lundholm *et al*.,[28] carried out a study to evaluate whether daily insulin treatment for weight-losing cancer patients attenuates the progression of cancer cachexia and improves metabolism and physical functioning in palliative care. One hundred and thirty-eight unselected patients with mainly advanced gastrointestinal malignancy were randomized to receive insulin (0.11±0.05 units/kg/day) plus best available palliative support [anti-inflammatory treatment (indomethacin), prevention of anemia (recombinant erythropoietin), and specialized nutritional care (oral supplements+home parenteral nutrition)] according to individual needs. Control patients received the best available palliative support according to the same principles. Insulin treatment significantly stimulated carbohydrate intake, decreased serum-free fatty acids, and increased whole body fat, particularly in trunk and leg compartments, whereas fat-free lean tissue mass was unaffected. Insulin treatment improved metabolic efficiency during exercise, but did not increase maximum exercise capacity and spontaneous physical activity. The authors concluded that insulin is a significant metabolic treatment in multimodal palliation of weight-losing cancer patients.

#### Branched-chain amino acids

Branched-chain amino acids are neutral amino acids with interesting and clinically relevant metabolic effects: They interfere with brain serotonergic activity and inhibit the overexpression of critical muscular proteolytic pathways. The potential role of branched-chain amino acids as antianorexia and anticachexia agents was proposed many years ago, but only recent experimental studies and clinical trials have tested their ability to stimulate food intake and counteract muscle wasting in anorectic, weight-losing patients.[29] In experimental models of cancer cachexia, BCAAs were able to induce a significant suppression in the loss of body weight, producing a significant increase in skeletal muscle wet weight[30] as well as in muscle performance and total daily activity.[31]

#### Oxandrolone

Recently, a prospective, randomized, phase III trial comparing the effects of oxandrolone (10 mg bid) and MA (800 mg q.d.) on weight, body composition, and quality of life (QOL) in 155 adult patients with solid tumors and weight loss receiving chemotherapy demonstrated that patients treated with oxandrolone experienced an increase in LBM, a reduction in fat mass, and reduced self-reported anorectic symptoms.[32]

#### Olanzapine

Olanzapine, an atypical neuroleptic with safe therapeutic window for several psychotic diseases, induces significant weight gain and positive metabolic effects. Preliminary data from a phase I pilot study by Braiteh *et al*.,[33] suggest that lower doses of OAZ are very well tolerated with promising clinical activity on weight, nutrition, and function in advanced cancer patients with cachexia. The trial is ongoing.

### Combined approach

From all the data presented, one can speculate that one single therapy may not be completely successful in the treatment of cachexia. From this point of view, treatments involving different combinations are more likely to be successful.[34] Cerchietti *et al*.,[35] carried out a pilot study using a multitargeted therapy in a homogeneous group of 15 lung adenocarcinoma patients, with evidence of ‘Systemic Immune-Metabolic Syndrome (SIMS)’ defined by authors as a particular variety of distressing systemic syndrome characterized by dysregulation of the psycho-neuro-immune endocrine homeostasis, with overlapping clinical manifestations: SIMS was defined as the presence of weight loss, anorexia, fatigue, performance status ≥2, and acute-phase protein response. Patients received MPA (500 mg twice daily), celecoxib (200 mg twice daily), plus oral food supplementation for 6 weeks. The results suggest that patients with advanced lung adenocarcinoma, treated with MPA, celecoxib, and dietary intervention, might have considerable improvement in certain SIMS outcomes. In a subsequent study, Cerchietti *et al*.,[36] aimed to test the hypothesis that by modulating systemic inflammation through an eicosanoid-targeted approach, some of the symptoms of the SIMS could be controlled. Twelve patients were evaluated for compliance and were assigned to one of the four treatment groups (15-, 12-, 9-, or 6-g dose, fractionated every 8 h). For patients assigned to 15- and 12-g doses, the overall compliance was very poor and unsatisfactory for patients receiving the 9-g dose. The maximum tolerable dose was calculated to be around two capsules tid (6 g of fish oil per day). In consequence, this dose was used for the randomized trial. Then, a second cohort of 22 patients with advanced lung cancer and SIMS were randomly assigned to receive either fish oil, 2 g tid, plus placebo capsules bid (*n*=12) or fish oil, 2 g tid, plus celecoxib 200 mg bid (*n*=10). All patients in both groups received oral food supplementation. After 6 weeks of treatment, patients receiving fish oil+placebo or fish oil + celecoxib showed significantly more appetite, less fatigue, and lower C reactive protein (C-RP) values than their respective baselines values (*P*<0.02 for all the comparisons). Additionally, patients in the fish oil+celecoxib group also improved their body weight and muscle strength compared to baseline values (*P*<0.02 for all the comparisons). Comparing both groups, patients receiving fish oil + celecoxib showed significantly lower C-RP levels (*P* = 0.005, *t*-test) and higher muscle strength (*P*=0.002, *t*-test), and body weight (*P*=0.05, *t*-test) than patients receiving fish oil+placebo. The addition of celecoxib improved the control of the acute-phase protein response, total body weight, and muscle strength. In the context of combined approaches, one of the most intriguing ones was our open phase II trial.[37,38] The aim of the study was to test the safety and efficacy of an integrated treatment based on diet, pharmaconutritional support administered per os, and drugs in a population of advanced cachectic cancer patients with cancer at different site. The following variables were assessed: clinical response, nutritional and functional variables, laboratory variables (as indicators of CACS/OS), and QOL, particularly fatigue. The ultimate goal of our study was that of translating the results obtained on CACS/OS symptoms found in advanced cancer patients into a prevention trial in a population of individuals at risk of developing CACS/OS. The treatment consisted of diet with high polyphenols content (400 mg), antioxidant treatment (300 mg/day alpha-lipoic acid+2.7 g/day carbocysteine lysine salt+400 mg/day vitamin E+30,000 IU/day vitamin A+500 mg/day vitamin C), and pharmaconutritional support enriched with two cans per day (n–3)-PUFA (eicosapentaenoic acid and docosahexaenoic acid), 500 mg/day medroxyprogesterone acetate, and 200 mg/day selective cyclooxygenase-2 inhibitor celecoxib. The treatment duration was 4 months. Thirty nine patients completed the treatment and were assessable. Body weight increased significantly from baseline as did LBM and appetite. There was an important decrease of proinflammatory cytokines IL 6 and tumor necrosis factor-α, and a negative relationship worthy of note was only found between LBM and IL-6 changes. As for quality of life, there was a marked improvement in the European Organization for Research and Treatment of Cancer QLQC30, Euro QL-5D(VAS), and fatigue assessed by MFSI-SF scores. The results have demonstrated the treatment to be both safe (without significant adverse events) and effective in 17 patients and highly effective in five patients as for increase of body weight, increase of LMB, decrease of proinflammatory cytokines, improvement of quality of life parameters, amelioration of fatigue symptom. Overall, these trials based on a combined approach, although supported by a good rationale, are all phase II studies enrolling a small number of patients and hence are to be considered preliminary and warrant further confirmation in phase III studies. On the basis of these results, we started a phase III randomized clinical trial to establish which was the most effective and safest treatment of CACS and oxidative stress in improving selected key variables as primary endpoints: increase of LBM, decrease of REE, increase of total daily physical activity, decrease of IL-6 and tumor necrosis factor-α, and improvement of fatigue. All patients were given basic treatment polyphenols plus antioxidant agents alpha-lipoic acid, carbocysteine, and vitamins A, C, and E, all orally administered. Patients were then randomized to one of the following five arms: (1) medroxyprogesterone acetate (500 mg/day)/megestrol acetate (320 mg/day), (2) pharmacologic nutritional support containing eicosapentaenoic acid (2 g/day), (3) L-carnitine 6 (4 g/day), (4) thalidomide (200 mg/day), or (5) medroxyprogesterone acetate/megestrol acetate plus pharmacologic nutritional support plus L-carnitine plus thalidomide. Treatment duration was 4 months. The sample size was 475 patients. The different single agents were selected on the basis of the following rationale. The antioxidant agents were shown to be effective in our previous studies.[39–44] The polyphenols, in particular quercetin, were included for their high activity as antioxidants.[45] Synthetic progestogens, MA/MPA, are currently the only approved drugs for CACS in Europe. Several randomized studies in mixed groups of weight-losing patients with cancer have suggested that MA/ MPA improves appetite and stabilizes weight to an extent greater than placebo.[4,46–49] The ω-3 polyunsaturated fatty acids (EPA and docosahexaenoic acid) have been shown to inhibit the production of proinflammatory cytokines and thereby to act positively on cancer cachexia. In experimental tumor models, EPA has demonstrated antitumor and anticachectic effects. Studies on weight-losing patients with pancreatic cancer receiving EPA have shown suppression of IL-6 production by peripheral blood mononuclear cells.[50–52] Barber *et al*.,[53] demonstrated that an EPA-enriched supplement added to the diet may reverse cachexia in patients with advanced pancreatic cancer. A double-blinded randomized study[15] in 200 patients with pancreatic cancer demonstrated a significant positive correlation between the assumption of the nutritional supplement and the increase of weight and LBM, provided EPA supplementation was ≥1.5 cartons a day. Carnitine is a cofactor required for transforming the free long-chain fatty acids into acyl-carnitine and for their subsequent transport into the mitochondrial matrix to produce acetyl-coenzyme A through the β-oxidation pathway. The relation between coenzyme A and carnitine is pivotal for cell energy metabolism: Coenzyme A is required for β-oxidation, metabolism of several amino acids, pyruvate dehydrogenase synthesis, and thus for triggering the tricarboxylic acid cycle.[17,54] Patients with cancer are especially at risk for carnitine deficiency; they frequently present with decreased caloric intake, and numerous antineoplastic drugs can interfere with the absorption and synthesis of carnitine. Thalidomide has multiple immunomodulatory and anti-inflammatory properties; its inhibitory effect on TNF-α and IL-6 production may be responsible for its anticachectic activity. Thus, thalidomide has been used for treatment of cachexia associated with acquired immunodeficiency syndrome, tuberculosis, and cancer. An interim analysis on 125 patients was published,[55] and the results obtained thus far, although to be considered with caution, seem to suggest that the most effective treatment for cancer-related anorexia/cachexia syndrome should be a combination regimen. However, the study is still in progress until completion of a planned accrual. In summary, there is not yet a consolidated treatment for cancer cachexia. As progestagens and corticosteroids, the only approved drugs for CACS, are only partially effective; research interest is currently shifting toward the use of different approaches addressing the potential targets involved in the pathophysiology of CACS.

### Future trends

Current new trends include anti-IL-6 humanized monoclonal antibody which in murine models appear to inhibit cancer cachexia;[56] IL-15, a cytokine expressed in skeletal muscle, is able to suppress the increased DNA fragmentation associated with muscle wasting in tumor-bearing rats[57] and also to have muscle anabolic effects *in vitro* and slow muscle wasting in rats with cancer cachexia.[58] Formoterol, a β2-adrenergic agonist, is a very efficient agent preventing muscle weight loss in tumor-bearing rats.[59] Recently, several promising androgen analogs have been developed, as potential selective androgen receptor modulators (SARMs), which claim to preferentially act on skeletal muscle. They bind to the androgen receptor (AR) with high affinity and exert strong pharmacological activity in selective tissues, although the mechanism is not well understood. In cellular and animal models, androgen-activated AR modulates myoblasts proliferation, promotes sexual dimorphic muscle development, and alters muscle fiber type. In the clinical setting, administration of anabolic androgens can decrease cachexia and speed wound healing.

A new class of nonsteroidal SARMs is being developed for use in cancer cachexia. SARMs are designed to have predominantly anabolic activity in muscle and bone with minimal androgenic effects in most other tissues. Evans *et al*.,[60] carried out a randomized phase II proof of concept study of Ostarine, the first-in-class SARM, in healthy postmenopausal women and elderly men prior to initiating a phase II study in cancer patients. The primary end point was change from baseline to 3 months in total LBM measured by dual energy X-ray absorptiometry. Ostarine was shown to improve LBM and physical performance in healthy older men and women. Ostarine had no unwanted androgenic side effects. A phase II study is planned to evaluate the safety and efficacy of Ostarine in patients with cancer cachexia. Myostatin has been implicated in several forms of muscle wasting, including cancer cachexia. Anti-myostatin strategies are, therefore, promising and should be considered in future clinical trials involving cachectic patients.[61] Recent experiments have shown that blockade of melanocortin signaling using antagonists to the melanocortin MC4 receptor attenuates disease-associated anorexia and wasting in rodent models of cancer and renal failure.[62] Predictive or early biomarkers of cachexia could be developed, which would aid in the selection of patients for early therapeutic intervention.[63] Key defining features of cachexia in humans (weight loss, reduced food intake, and systemic inflammation) now provide not only a framework for classification but also a rationale for targets for therapeutic intervention. The role of age and immobility in muscle wasting also provides a rationale for the nature of nutritional support in cachexia. Multimodal approaches that address these key issues can stabilize and even improve the nutritional status, function, and quality of life of at least a proportion of cachectic cancer patients. The current evidence justifies new enthusiasm for the design of complex intervention studies in the management of cancer cachexia.[64]

In summary, based on current views on the cachexia syndrome in cancer patients, we put forward the following recommendations:

1. Wasting is a predictable event in many cancer patients, readily diagnosed by assessment of weight, change in appetite, and food intake. Often these patients will also have anemia and low albumin, with a concomitant increase in C-reactive protein. The above simple assessments should form a consistent part of the record of all advanced cancer patients.
2. Use a systematic formal guide to rule out treatable secondary causes of wasting.
3. At the onset and throughout the course of illness, offer patients nutritional counseling (they should have access to a nutrition team with a special interest in wasting disorders), encourage them to take part in a rehabilitation program tailor made for their needs and abilities, and consider the use of specific nutraceutical and pharmacologic interventions. Follow-up visits should not only note careful evaluation of antitumor therapy and tumor volume, but also regular assessment of symptom control, weight, appetite, and function.
4. Take careful note of the full medication profile of patients who are wasting. These might include drugs that could have a favorable effect on cachexia (cardiac agents such as the statins, ACE inhibitors) and other agents that may be deleterious (e.g. herbal medications laced with corticosteroids).
5. Testosterone status should be established in cancer patients with the cachexia syndrome. If clearly reduced, physiologic testosterone supplementation should be considered after discussion with the patient.
6. Patients must be assured of a reasonable intake of amino acids. Protein-containing foods are indicated and rich sources of both essential and nonessential amino acids will support any anabolic potential.
7. Clinical researchers should be more cognizant of the work of their colleagues in sports medicine, AIDS, and geriatrics. Learning from their enterprises, further studies on creatine and supraphysiologic amounts of amino acids with a particular role in protein synthesis should be conducted. Similarly, the role of supraphysiologic doses of anabolic agents, in combination with nutrients and compounds that control muscle proteolysis, should receive high priority.
8. There are few, if any, negative exercise trials. Patients should be encouraged to keep active or take part in tailored exercise programs, and studies on nutritional and pharmacologic agents should incorporate the potential additive effects of exercise.
